# Supplementary material for: The importance of physical performance in the assessment of patients on haemodialysis: A survival analysis
Source: PLoS One. 2022 May 19;17(5):e0268115. doi: 10.1371/journal.pone.0268115 (PMC9119466; doi:10.1371/journal.pone.0268115)
Supplement: S1 Table — (DOCX) [file pone.0268115.s001.docx]

# S1 Table. Dialysis fall risk index.

| **Topic** | **Check** | | | **Score** |
| --- | --- | --- | --- | --- |
| Age | ≥80 years old | | |  |
|  | - Yes | | | 1.5 |
|  | - No | | | 0 |
| Serum C-reactive Protein | >2.9 mg/dL | | |  |
|  | - Yes | | | 2.0 |
|  | - No | | | 0 |
| Risk for malnutrition | <24 on 30 | | |  |
|  | - Yes | | | 0.5 |
|  | - No | | | 0 |
| Physical examinations | Standing balance | | |  |
|  | - Side-by-side stand | | |  |
|  | - 10 sec | - 1 |  |  |
|  | - <10 sec | - 0 |  |  |
|  | - Semi-tandem stand | | |  |
|  | - 10 sec | - 1 |  |  |
|  | - <10 sec | - 0 |  |  |
|  | - Full-tandem stand | | |  |
|  | - 10 sec | - 2 | - <8 points | 2.5 |
|  | - 3-9.9 sec | - 1 |  |  |
|  | - <3 sec | - 0 | - 9-10 points | 2.0 |
|  | 6MWT |  |  |  |
|  | - >350 m | - 4 | - 11-12 points | 0 |
|  | - 300-350 m | - 2 |  |  |
|  | - <300 m | - 0 |  |  |
|  | STS |  |  |  |
|  | - <11.19 sec | - 4 |  |  |
|  | - 11.20-13.69 sec | - 3 |  |  |
|  | - 13.70-16.69 sec | - 2 |  |  |
|  | - 16.70-49.99 sec | - 1 |  |  |
|  | - >50 sec | - 0 |  |  |
| Handgrip strength | Male <26kg, female <18kg |  |  |  |
|  | - Yes |  |  | 1.5 |
|  | - No |  |  | 0 |
| Intra-dialytic hypotension | MAP decrease >9.99 mmHg |  |  |  |
|  | - Yes |  |  | 1.5 |
|  | - No |  |  | 0 |
| Risk of Falls assessment | Tinetti <11 on 12 points |  |  |  |
|  | - Yes |  |  | 2.5 |
|  | - No |  |  | 0 |
| Total score |  |  |  | /12 |
| Abbreviations: 6MWT, six-minute walking test; MAP, mean arterial pressure; STS, sit-to-stand test | | | | |
