# Supplementary material for: The importance of physical performance in the assessment of patients on haemodialysis: A survival analysis
Source: PLoS One. 2022 May 19;17(5):e0268115. doi: 10.1371/journal.pone.0268115 (PMC9119466; doi:10.1371/journal.pone.0268115)
Supplement: Attachment — Submitted filename: comments.doc [file pone.0268115.s003.doc]

Overall, the idea of research is very interesting to be studied nowadays and paper is coherently developed. However, there are some comments and suggestions.

**Abstract**

Page 2 line 21: add ''Aim'' before (To assess the association between different 22 measures of physical performance and survival in dialysis patients).

Page 2 line 26: write the full name of (FICSIT-4)

Page 2 line 32: write the full name of (HR)

Keywords: write it in alphabetical order

**Introduction**

Line 53 to 64, please insert the reference for this section

**Materials and methods**

Study design is not mentioned

Correct the title on page 4 line 76 (Materials an& methods) to (Materials and methods)

Page 5 line 103: please clarify the (Quadriceps peak torque) test protocol as did you examine eccentric or concentric Quadriceps peak torque, the knee at angular velocity

Validity and reliability of the used instruments used in this study should be clarified

**Statistical analysis**

Page 7 line 163 the authors mentioned the reference Fine and Gray [23, 24]. However 23 and are two different references. Correct

Delete the word abbreviation bellow all tables

**Funding**

Page 17 line 372 and 373 include funding and conflicts of interest. Please write in separate

**References**

Update some references is required
