# Supplementary material for: The importance of physical performance in the assessment of patients on haemodialysis: A survival analysis
Source: PLoS One. 2022 May 19;17(5):e0268115. doi: 10.1371/journal.pone.0268115 (PMC9119466; doi:10.1371/journal.pone.0268115)
Supplement: Attachment — Submitted filename: Response to Editor and Reviewers.docx [file pone.0268115.s004.docx]

**Revision: manuscript PONE-D-20-37730**

**The importance of physical performance in the assessment of patients on haemodialysis: a survival analysis**

Dear Editor,

We would like to thank you for the opportunity to improve our work and to give us the chance to resubmit the manuscript to PLOS ONE. We are grateful for the editorial and reviewer comments and thank them for their appreciation.

The formulated comments are well taken; below you will find point-by-point responses to the comments and questions raised in the decision letter. Stated citations (page; line) refer to the revised version of the manuscript with track changes.

Yours sincerely,

Karsten Vanden Wyngaert

1. EDITORIAL COMMENTS

# EDITOR COMMENTS

**1) 1. Please ensure that your manuscript meets PLOS ONE's style requirements, including those for file naming.**

We adapted the manuscript to meet PLOS ONE’s style requirements. The guidelines for file naming were followed and the necessary adaptations were made.

2. RESPONSE TO REVIEWERS

# REVIEWER #1

**This paper presents a reasonable, if incomplete, analysis of a set of predictor markers for quality of life and survival. Overall many of the methods are sounds and of reasonable sophistication. However, major concerns still remain.**

We thank the reviewer for his/her constructive criticism and interest in our work.

**1) In assessing categorical outcomes (survival or hospitalization, in particular) it is generally preferred to fit a model based on a benchmark time point (i.e., 1 year).**

Indeed, a benchmark of one year is an often used time point when examining categorical outcomes (such as incidence data). Nevertheless, when performing a survival analysis, a time-to-event analysis can be preferred because it results in more reliable effect sizes. Also, due to the relatively small sample size, the number of events – and thereby the power and appropriateness to use covariates in the analysis such as age and sex– was too low when considering the cut-off point of 1 year. Accordingly, we preferred to report a time-to-event analysis, which method has been frequently used in literature as well (*Ebrahimi et al., Factors influencing survival time of hemodialysis patients; time to event analysis using parametric models: a cohort study. BMC Nephrol 20, 215* & *Zheng C, Tian J, Wang K, Han L, Yang H, Ren J, Li C, Zhang Q, Han Q, Zhang Y. Time-to-event prediction analysis of patients with chronic heart failure comorbid with atrial fibrillation: a LightGBM model. BMC Cardiovasc Disord. 2021 Aug 4;21(1):379.*).

**2) Additionally, inferential results (p-values, confidence intervals, etc.) are listed in a haphazard manner among the results, with some inferential findings missing completely.**

We thank the reviewer for this remark, which will improve the transparency of our manuscript and agree that the reporting of inferential results could and should be improved in our manuscript. The following inferential results were added and adapted to the body of the results section of the revised version of the manuscript.

Results, Page 10, lines 195-197: “This study included 117 (low-/high-care centres n=54/n=63) patients on maintenance haemodialysis (average 68 years ± 16; 41% female) and obtained data on their physical performance, mortality, comorbidity, and hospitalisation.”

Results, Page 11, line 218-222: “The risk of falls by DFRI was associated with an increased mortality risk (HR = 20.4, p=0.003). A walking distance of less than 298 meters (specificity = 0.583; sensitivity = 0.889, Fig. 1) was associated with an increased mortality risk (HR = 7.4, p=0.045).”

Results, Page 11, line 224-226: “After adjusting for age, sex, comorbidity and dialysis vintage, each increase on the 6MWT (m) was associated with a 0.4% decrease in mortality risk (HR = 0.996, 95%CI [0.994; 0.998], p<0.001, Table 4).”

**3) Finally, the purpose of the study should be listed more clearly. The use of the 6MWT assessment tool is justified, but the multi-variate modelling procedure is incomplete and missing useful details about the prediction of outcomes. This is especially important given the disparity between significance in the univariate and multivariate analyses.**

Thank you for this comment. We adapted the aim of the study in the revised version of the introduction to make it more transparent. The main aim of this study was to examine which measures of physical performance are related to survival and hospitalisation in patients on haemodialysis. This research question was answered by the univariate analysis. Additionally, we also aimed to identify the most prominent measure of physical performance. This second research question was analysed via a backward stepwise regression (multivariate) model. Whereas these two aims were not clearly reported, we adapted the following sentences:

Introduction, page 5, lines 75-80: “The aim of this study was to identify measures of physical performance that are associated with survival and hospitalisation in patients on haemodialysis. Additionally, we aimed to examine which measure(s) of physical performance was/were the most prominent in this association and could, accordingly, be relevant to be used in the clinical screening of patients on haemodialysis.”

Additionally, we added an explanation of which statistical methods were used to answer the main and secondary research question. The following sentences were added to the revised version of the Statistical Analysis:

Materials and methods, page 9, lines 182-186: “Also, the unique associations between measures of physical performance and survival were analysed via a univariate analysis. A multivariate stepwise backward regression model was used to examine which measure(s) of physical performance was/were most prominent in the association between physical performance and survival.”

**More specific comments are given below:**

**4a) Line 30: The 15/100 patient deaths per year is plausible but should either be presented as a percentage or in terms of the total number of patient-years.**

Thank you for this suggestion. We adapted the sentence.

The following sentences were adapted in the revised version of the manuscript

Abstract, page 2, lines 30-31: “During the observation period (median 33, min 30 max 45 months), 45 patients died (=38.5%), resulting in a mortality rate of 15% per year.”

Results, page 10, line 200-201: “This resulted in a mortality rate of 15%.”

**4b) Line 33-36: It is not obvious why there are no inferential descriptors (p-values, confidence intervals) in this set of results.**

We agree with the reviewer that some important inferential descriptors were lacking in the abstract. We also agree that this information is important for the readers to interpret the abstract. Accordingly, we added information on the inferential descriptors to the abstract.

The following sentences were adapted and added to the revised version of the abstract:

Abstract, page 2, lines 34-40: “A score lower than 298 meters (specificity = 0.583; sensitivity = 0.889) on the 6MWT was established as a haemodialysis-specific cut-off point for mortality risk. Each increase in 6MWT (m) corresponded with a 0.4% decrease in mortality risk (HR = 0.996, 95%CI [0.994; 0.998]). The 6MWT as also associated with comorbidity (F-value = 6.1, p=0.015).”

**4c) Line 78: No description is found about the effect of centre on the results. At the least, the number of patients contributed by each centre should be given.**

Thank you for this remark. Patients were recruited from 2 high-care dialysis units (Ghent n=41 and Antwerp n=22 patients) and 5 low-care dialysis units (Ghent n=10 ; Aalst n=19 ; Geraardsbergen n=5 ; Wetteren n=7 ; and Antwerp n=13). Note that the dialysis units in Aalst, Geraardsbergen, and Wetteren are satellite units of Ghent University Hospital, resulting in an Antwerp/Ghent recruitment-ratio of 35/82. Data regarding the selection process and the high-care/low-care ratio (i.e. 63/54) was added to the revised version of the manuscript.

Since Antwerp and Ghent are cities that are a 35-minutes’ drive away from each other, and no differences in standard care or representative cohort characteristics are expected, we decided to report the number of patients recruited from low-care versus high-care dialysis centres, because this information is an important characteristic of our cohort.

The following information was added to the revised version of the manuscript:

Results, page 10, lines 195-197: “This study included 117 (low-/high-care centres n=54/n=63) patients on maintenance haemodialysis (average 68 years ± 16; 41% female) and obtained data on their physical performance, mortality, comorbidity, and hospitalisation.”

**4d) Line 93: If the sequence of physical examinations was indeed randomized, then more detail is required. Discussion of how the randomization was done and the relative effect of the random order would be important.**

We used opaque envelopes to randomise the sequence of examinations. The requested information was added to the manuscript.

The following information was added to the revised version of the manuscript:

Methods, page 6, lines 101-103: “The sequence of physical examinations was randomized using opaque envelopes and a minimum 3-minute interval between tests was respected. This 3-minute interval was used to let patients recover from previous examinations and to avoid bias.”

**4e) Line 134: This sentence is not clearly written. It appears that you did not exclude patients who received transplants.**

Thank you. Patients that were transplanted during the follow-up time were censored, via the appropriate analysis (i.e. Competing Risk). They were not excluded from the baseline data as this would induce a selection bias. Also patients that had a failed transplant were censored. I adapted the sentence to improve the transparency of this study.

The following information was added to the revised version of the manuscript:

Methods, page 8, lines 148-150: “Transplantation during the study period was no exclusion criteria in the final survival-analyses as this would introduce censoring bias [29], they were however considered as a competing risk of survival and therefore censored via the appropriate analysis.”

**4f) Line 148: The contributed libraries used in this analysis should also be listed.**

Thank you for this suggestion. The libraries were added to the Statistical Analysis.

The following information was added:

Material and methods, page 8, lines 163-165: “Data was analysed using R statistics software version 3.5.2 (contributed libraries: *foreign*, *survival* and *cmprsk*) and SAS statistical software version 9.4 (SAS institute Inc.; Cary, NC, USA).”

**4g) Lines 153-157: It appears that the ROC curve was used to assess a cut-off for using 6MWT as a predictor of mortality. It is not clear how the area under the curve was instrumental, unless other models or predictors were used, in which case they should be described. Given the ROC curve described, it is not clear, based on the description, how the cut-off point was chosen.**

Thank you for this suggestion to improve the transparency of our manuscript. We used the Youden’s Index to calculate the cut-off point on the ROC. This point is the maximum point of = sensitivity+specificity – 1. The area under the curve was only calculated to identify models that were clinically relevant.

The following information was added to the revised version of the manuscript:

Materials and methods, page 9, lines 172-174: “Accordingly, based on the Youden’s index, a 298 m cut-off point (specificity = 0.583; sensitivity = 0.889) on the 6MWT test was established and used in the survival analysis (Fig. 1).”

**4h) Line 166: It appears that the authors mean “generalized linear models”.**

Yes, we indeed wanted to refer to generalized lineal models. Thank you for addressing this mistake.

The following sentence was adapted:

Materials and methods, page 9, lines 186-188: “Next, generalized linear models were used to evaluate the association between hospitalisation and comorbidity on the one hand and the different measures of physical performance on the other.”

**4i) Line 168: Stepwise methods or the variety of penalized regression modelling techniques are preferable modeling approaches to pulling out significant variables in a univariate analysis.**

Thank you for this suggestion. We are aware of these approaches to pull out variables in a univariate analysis. We actually did perform a (backward) stepwise regression analysis. This description was added to the manuscript. We also agree with the reviewer that a penalized model technique might even be a more solid technique. This method (e.g. a ridge regression analysis), however, is complicated and often not understood by readers. We already performed such a penalized technique in previously published articles, and were confronted with the feedbacks that a simple statistical approach is often favoured over a complicated approach because it improves the transparency of the study (*Vanden Wyngaert, K.; Van Craenenbroeck, A.H.; Holvoet, E.; Calders, P.; Van Biesen, W.; Eloot, S. Composite Uremic Load and Physical Performance in Hemodialysis Patients: A Cross-Sectional Study. Toxins 2020, 12, 135.*). Therefore, we decided to stick with our backward regression analysis and to not use a penalized technique, especially because a stepwise regression method has been considered a valid method as well. Nevertheless, we would like to thank the reviewer for this comment because it improves the transparency of our manuscript.

Materials and methods, page 9, lines 182-186: “Also, the unique associations between measures of physical performance and survival were analysed via a univariate analysis. A multivariate stepwise backward regression model was used to examine which measure(s) of physical performance was/were most prominent in the association between physical performance and survival.”

**4j) Line 169: Based on the results (comparing inferences in the univariate and multivariate models), it appears that more collinearity was a bigger issue than reported. The results do not make clear why the results are so divergent.**

The reviewer is right to state that there seems to be some remaining collinearity present. Measures of physical function are related to each other, and collinearity between the different measures of physical performance is evident. However, we used a reliable tool to measure collinearity between de different measures of physical function and, subsequently, excluded the Dialysis Fall Risk Index (DFRI) from the analysis. To meet with the reviewer’s remark, we added the presence of a presumed collinearity to the limitations of the study.

The following sentence was added to the limitations:

Discussion, page 18, lines 375-377: “Second, although we excluded variables that caused multicollinearity into the statistical models, some collinearity between the remaining measures of physical function may still be present.”

**4k) Lines 196/198: The change in mortality risk should be given in terms of the statistics they were based upon.**

In order to improve the transparency of the results and to improve the consistency in reporting style, we adapted the following sentences in the revised version of the manuscript:

Results, Page 11, line 218-222: “The risk of falls by DFRI was associated with an increased mortality risk (HR = 20.4, p=0.003). A walking distance of less than 298 meters (specificity = 0.583; sensitivity = 0.889, Fig. 1) was associated with an increased mortality risk (HR = 7.4, p=0.045).”

**4l) Lines 200/202: The significance of 6MWT in this setting is surprising and difficult to believe, given how close it is to one. Standard errors or confidence intervals might help here.**

Indeed, adding the confidence intervals would help the readers to interpret this result. The 95%CI was [0.994; 0.998]. I added this information to the manuscript.

The following information was added to the revised version of the manuscript:

Results, Page 11, line 224-226: “After adjusting for age, sex, comorbidity and dialysis vintage, each increase on the 6MWT (m) was associated with a 0.4% decrease in mortality risk (HR = 0.996, 95%CI [0.994; 0.998], p<0.001, Table 4).”

This information was also added to the abstract in the revised version of the manuscript:

Abstract, page 2, lines 36-38: “Each increase in 6MWT (m) corresponded with a 0.4% decrease in mortality risk (HR = 0.996, 95%CI [0.994; 0.998]).”

**4m) Line 202: It is not clear how a hazard ratio of 0.996 translates into a 4% change in mortality risk.**
Thank you for this comment. The reviewer is correct that HR = 0.996 does not translate in a 4% change in mortality risk. I adapted this information in the revised version of the manuscript.

Results, Page 11, line 224-226: “After adjusting for age, sex, comorbidity and dialysis vintage, each increase on the 6MWT (m) was associated with a 0.4% decrease in mortality risk (HR = 0.996, 95%CI [0.994; 0.998], p<0.001, Table 4).”

**4n) Line 276-277: The robustness of the cut-off is not justified, since there is no obvious attempt to assess the factor 6MWT as it interacts with the covariates listed.**

Thank you for this comment. Unfortunately, we have to disagree with the reviewer. We do not examine a causal relationship, but merely a predictive one. In a model examining causal relationships, confounding factors may bias the causal relationship between two variables. In a prediction-model, however, confounding is in essence not relevant. The question is not whether the 6MWT is associated with other variables that are associated with mortality, but whether the 6MWT itself is predictor of mortality. Therefore, we believe that the possible interactions of the 6MWT with other variables is not important to this analysis.

# REVIEWER #2

**Overall, the idea of research is very interesting to be studied nowadays and paper is coherently developed. However, there are some comments and suggestions.**

**1) Abstract**

**1a) Page 2 line 21: add ''Aim'' before (To assess the association between different 22 measures of physical performance and survival in dialysis patients).**

Thank you for this suggestion.

The following sentence was adapted:

Abstract, page 2, lines 21-22: “We aimed to assess the association between different measures of physical performance and survival in dialysis patients.”

**1b) Page 2 line 26: write the full name of (FICSIT-4).**

We adapted this sentence.

Abstract, page 2, line 26: “Muscle strength (quadriceps, handgrip strength, and sit-to-stand), exercise capacity (six-minute walking test, 6MWT) and the risk of falls (Dialysis Fall Index, Tinetti, and Frailty and Injuries: Cooperative Studies of Intervention Techniques) were measured at the time of inclusion.”

**1c) Page 2 line 32: write the full name of (HR)**

Thank you for this remark. We wrote HR in full the first time.

Abstract, page 2, lines 32-34: “All domains of physical performance were associated with mortality, with the highest hazards for an increased risk of falls (Hazard Ratio (HR) = 20.4, p=0.003) and poor exercise capacity (HR = 7.4, p<0.001).”

**1d) Keywords: write it in alphabetical order**

The order was adapted as suggested by the reviewer

Indexing Key Words, page 3, lines 45-46: “Cardiovascular disease; end-stage kidney disease; physical performance; morbidity; mortality”

**2) Introduction**

**2a) Line 53 to 64, please insert the reference for this section**

We would like to thank the reviewer for this remark. There are indeed two references lacking in the section. The study by Caspersen et al., is a key publication regarding terminology and should be added to the introduction.

The following references were added to the introduction:

Introduction, Page 4, lines 58-69: “Physical performance is an umbrella term that represents the different domains involved when performing physically challenging activities such as walking or daily chores. In general, it comprises the ability to make movements by producing muscle activity (muscle strength), the capacity to perform these movements continuously for a longer period (exercise capacity), and to exert the necessary control and coordination of movements. These domains can be assessed in a purely analytical way (e.g. quadriceps peak torque at knee 90° flexed, maximal muscle strength) or by simulating activities of daily living (e.g. getting up from a seating position, functional lower limb muscle strength) [4]. The fear of falls is an important cause of a sedentary lifestyle and physical deterioration as well. An increased risk of falls has a multifactorial aetiology in patients on haemodialysis, often involving both domains of physical performance and impaired balance and coordination; and assessment of the risk of falls should therefore be included in the physical screening of these patients [5].”

Reference: “4. Caspersen CJ, Powell KE, Christenson GM. Physical activity, exercise, and physical fitness: definitions and distinctions for health-related research. Public health reports (Washington, DC : 1974). 1985;100(2):126-31. PubMed PMID: 3920711.”

Reference: “5. Kono K, Nishida Y, Yabe H, Moriyama Y, Mori T, Shiraki R, et al. Development and validation of a Fall Risk Assessment Index for dialysis patients. Clinical and experimental nephrology. 2018;22(1):167-72. Epub 2017/06/22. doi: 10.1007/s10157-017-1431-8. PubMed PMID: 28634773.”

**3) Materials and methods**

**3a) Study design is not mentioned**

The study design was indeed lacking. This study reports longitudinal data. This information was added to the Materials and Methods.

Materials and methods, page 5, lines 92-94: “This longitudinal study was part of a project to identify potential (predicting) biomarkers based on functional capacity, nutritional status and/or QoL in patients on dialysis (registration number on clinicaltrial.gov: NCT03910426).”

**3b) Correct the title on page 4 line 76 (Materials & methods) to (Materials and methods)**

The title was adapted in the revised version of the manuscript.

**3c) Page 5 line 103: please clarify the (Quadriceps peak torque) test protocol as did you examine eccentric or concentric Quadriceps peak torque, the knee at angular velocity.**

Thank you for this pertinent remark. A description of the quadriceps peak torque assessments is indeed lacking. Patients had to be in a sitting position with 90° knee and 90° hip flexion. The hand-held dynamometer was placed on the anterior aspect of the tibia of the dominant leg, proximal to the ankle joint. The participants had to perform a voluntary maximal isometric contraction for five seconds against the dynamometer, which was fixated by the physiotherapist which applied enough resistance so movement of the limb was avoided and maximal strength was assessed (*Mentiplay BF, Perraton LG, Bower KJ, Adair B, Pua Y-H, Williams GP, et al. Assessment of Lower Limb Muscle Strength and Power Using Hand-Held and Fixed Dynamometry: A reliability and Validity Study. 2015*). The leg was tested three times with one minute rest in-between the assessments. The best out of the three attempts was noted. Verbal encouragement was given. A short description of the protocol was added to the revised version of the manuscript.

Materials and methods, page 6, lines 118-119: “Quadriceps strength was measured by maximal isometric contraction and patients had to be in a sitting position with 90° flexion in both knee and hip.”

**3d) Validity and reliability of the used instruments used in this study should be clarified**

Thank you for this methodological suggestion. We added the validity and reliability of the used instruments to materials and methods.

The following information was added to the revised version of the manuscript:

Materials and methods, page 6, lines 113-118: “Maximal muscle strength. Quadriceps peak torque (Microfet; Biometrics, Almere, the Netherlands) (Intraclass Correlation Coefficient (ICC) ranging between 0.76 and 0.96) [13, 14] and handgrip strength (JAMAR Hydraulic Hand Dynamometer; Patterson, Nottinghamshire, United Kingdom) (ICC = 0.93) [15] were measured by hand-held dynamometers according to the guidelines of Bohannon [16] and American Society of Hand Therapists [17] respectively.”

Materials and methods, page 7, lines 123-125: “The five repetition Sit-to-Stand test (STS) was used to examine functional lower limb muscle strength and was expressed as the time patients needed to stand up 5 times from a seating position (ICC = 0.97) [19, 20].”

Materials and methods, page 7, lines 129-131: “The six-minute walking test (6MWT) is considered the gold standard for assessing functional exercise capacity and was performed following the American Thoracic Society guidelines (ICC > 0.90) [22, 23]”

Materials and methods, page 7, lines 140-142: “Next, the Tinetti (ICC > 0.8) [25] and FICSIT tools (Frailty and Injuries Cooperative Studies of Intervention Technique) (ICC = 0.79) [26] were used to examine gait dysfunctions and static balance [27].”

The following references were also added to the revised version of the manuscript:

References: “13. Mentiplay BF, Perraton LG, Bower KJ, Adair B, Pua YH, Williams GP, et al. Assessment of Lower Limb Muscle Strength and Power Using Hand-Held and Fixed Dynamometry: A Reliability and Validity Study. PloS one. 2015;10(10):e0140822. Epub 2015/10/29. doi: 10.1371/journal.pone.0140822. PubMed PMID: 26509265; PubMed Central PMCID: PMCPMC4624940.”

References: “14. Vanpee G, Hermans G, Segers J, Gosselink R. Assessment of limb muscle strength in critically ill patients: a systematic review. Critical care medicine. 2014;42(3):701-11. Epub 2013/11/10. doi: 10.1097/ccm.0000000000000030. PubMed PMID: 24201180.”

References: “15. Hermans G, Clerckx B, Vanhullebusch T, Segers J, Vanpee G, Robbeets C, et al. Interobserver agreement of Medical Research Council sum-score and handgrip strength in the intensive care unit. Muscle & nerve. 2012;45(1):18-25. Epub 2011/12/23. doi: 10.1002/mus.22219. PubMed PMID: 22190301.”

References: “20. Ng SS, Kwong PW, Chau MS, Luk IC, Wan SS, Fong SS. Effect of arm position and foot placement on the five times sit-to-stand test completion times of female adults older than 50 years of age. J Phys Ther Sci. 2015;27(6):1755-9. Epub 2015/07/17. doi:”

References: “23. Demers C, McKelvie RS, Negassa A, Yusuf S. Reliability, validity, and responsiveness of the six-minute walk test in patients with heart failure. American heart journal. 2001;142(4):698-703. Epub 2001/10/02. doi: 10.1067/mhj.2001.118468. PubMed PMID: 11579362.”

References: “25. Kegelmeyer DA, Kloos AD, Thomas KM, Kostyk SK. Reliability and validity of the Tinetti Mobility Test for individuals with Parkinson disease. Physical therapy. 2007;87(10):1369-78. Epub 2007/08/09. doi: 10.2522/ptj.20070007. PubMed PMID: 17684089.”

References: “26. Blankevoort CG, van Heuvelen MJ, Scherder EJ. Reliability of six physical performance tests in older people with dementia. Physical therapy. 2013;93(1):69-78. Epub 2012/09/15. doi: 10.2522/ptj.20110164. PubMed PMID: 22976448.”

**3e) Page 7 line 163 the authors mentioned the reference Fine and Gray [23, 24]. However 23 and are two different references. Correct**

Thank you for this remark. We corrected this mistake.

The following sentence was adapted:

Materials and methods, page 9, lines 178-180: “We applied a competing risk survival analysis (competing event: transplantation) to examine the association between the different measures of physical performance and mortality as described by Fine and Gray [31]”

**3f) Delete the word abbreviation bellow all tables**

The term ‘abbreviation’ was deleted below all tables.

**4) Funding**

**4a) Page 17 line 372 and 373 include funding and conflicts of interest. Please write in separate**
The statements were adapted.

The following information was adapted in the revised version of the manuscript:

Discussion, page 19, lines 396-401: “

Conflict of interest

There are no conflicts of interest.

Funding

This research did not receive any specific grant from funding agencies in the public, commercial, or not-for-profit sectors.”

**5) References**

**5a) Update some references is required**

Thank you for this remark. All references were checked and updated (including the newly added references).
