# Supplementary material for: The importance of physical performance in the assessment of patients on haemodialysis: A survival analysis
Source: PLoS One. 2022 May 19;17(5):e0268115. doi: 10.1371/journal.pone.0268115 (PMC9119466; doi:10.1371/journal.pone.0268115)
Supplement: Attachment — Submitted filename: Comments to authors.doc [file pone.0268115.s005.doc]

**Comments to authors**

Thank you for your effort to revise your manuscript. You have done great effort and responded to suggested comments. I have no further comments on your work.
