# Supplementary material for: The importance of physical performance in the assessment of patients on haemodialysis: A survival analysis
Source: PLoS One. 2022 May 19;17(5):e0268115. doi: 10.1371/journal.pone.0268115 (PMC9119466; doi:10.1371/journal.pone.0268115)
Supplement: Attachment — Submitted filename: Response to Reviewers.docx [file pone.0268115.s006.docx]

**Revision: manuscript PONE-D-20-37730R1**

**The importance of physical performance in the assessment of patients on haemodialysis: a survival analysis**

Dear Editor,

We would like to thank you again for the opportunity to improve our work and to give us the chance to resubmit the manuscript to PLOS ONE. We are grateful for the editorial and reviewer comments and thank them for their appreciation.

The formulated comments are well taken; below you will find point-by-point responses to the comments and questions raised in the decision letter. Stated citations (page; line) refer to the revised version of the manuscript with track changes.

Yours sincerely,

Karsten Vanden Wyngaert

1. EDITORIAL COMMENTS

# JOURNAL REQUIREMENTS AND EDITOR COMMENTS

**1) Please review your reference list to ensure that it is complete and correct. If you have cited papers that have been retracted, please include the rationale for doing so in the manuscript text, or remove these references and replace them with relevant current references. Any changes to the reference list should be mentioned in the rebuttal letter that accompanies your revised manuscript. If you need to cite a retracted article, indicate the article’s retracted status in the References list and also include a citation and full reference for the retraction notice.**

Thank you for the remark. We reviewed the reference list and confirm that all references are appropriate.

2. RESPONSE TO REVIEWERS

# REVIEWER #1

**The current version of this article represents a notable improvement over the initial version. However, concerns do remain.**

We thank the reviewer for his/her constructive criticism and observation of our effort to improve the manuscript according to the provided suggestions.

**1) The term "competing risk" should be modified to be "competing risks".**

Thank you for this linguistic suggestion.

The following sentences were adapted in the revised version of the manuscript:

Abstract, page 2, lines 26-27: “Data were analysed by general linear models and competing risks survival hazard models.”

Materials and methods, page 8, lines 145-147: “Transplantation during the study period was no exclusion criteria in the final survival-analyses as this would introduce censoring bias [29], they were however considered as a competing risks of survival and therefore censored via the appropriate analysis.”

Materials and methods, page 9, lines 175-177x: “We applied a competing risks survival analysis (competing event: transplantation) to examine the association between the different measures of physical performance and mortality as described by Fine and Gray [31].”

Results, page 11, lines 216-217: “All domains of physical performance were associated with survival in the univariate competing risks regression models (Tables 2 and 3).”

The following titles of Tables 2-5 were adapted in the revised version of the manuscript:

Results, page 12, line 231: “Table 2. Unadjusted competing risks regression model for mortality.”

Results, page 12, lines 236-237: “Table 3. Unadjusted competing risks regression model for mortality by the presence of impairments in physical performance.”

Results, page 12, line 243: “Table 4. Adjusted competing risks regression model for mortality.”

Results, page 13, lines 248-249: “Table 5. Adjusted multivariate competing risks regression model by the presence of impairments in physical performance.”

**2) A description of the rate of censoring should be provided. How many subjects dropped out early? What was the rate per annum? What was the median/maximum time of censoring?**

Based on your suggestion, we added extra data on the censoring of patients. The rate of censoring (i.e. censored population over the population with no observed event) was 18%. We added this information to the revised version of the manuscript. No patients dropped out early and, consequently, there was no lost-to-follow-up. We added this information as well to the manuscript. Additionally, the mortality rate per annum was 35.6% (16 patients), 33.3% (15 patients), 13.3% (6 patients), and 17.8% (8 patients) for the 1^st^ to 4^th^ annum. With respect to the competing risks, 6, 4, and 3 patients were transplanted in the first, second and third annum respectively. Also the minimum and maximum time of censoring was added (i.e. minimum time of censoring 49 days; maximum time 796 days).

The following information was added to the revised version of the manuscript:

Results, page 10, lines 196-203: “During an observation period of 33 months on average (ranging between 30 and 45 months), 45 subjects (38.5%) died and 13 patients (11.1%) were transplanted, which resulted in a mortality rate of 15% and rate of censoring of 18%. No patients dropped out during the observation period. Sixteen, fifteen, six, and eight patients died in the first, second, third, and fourth annum, which corresponds with a relative ratio of 35.6%, 33.3%, 13.3%, and 17.8%. With respect to the competing risks, six, four, and three patients were transplanted in the first, second and third annum respectively, resulting in a medium censoring time of 425 days, [minimum 49 days; maximum 796 days].”

**3) The use of a cutoff for 6MWT is confusing. The cutoff was (seemingly) based on a categorical analysis of overall mortality, as opposed to a benchmark level of mortality. Possible bias a result of censoring was not considered. Using mortality as an outcome both for finding a cutoff and assessing the hazard ratio risk brings bias both in to the estimation of the hazard ratio risk and the inference of a such a risk. Unless it is absolutely justified otherwise, the cutoff should be based on an optimization in the survival modeling space, based on a performance metric such as concordance. Additionally, the sensitivity of the optimal cutoff to population subset selection and/or variable should be included.**

We would like to thank the reviewer for this remark because the statistical methods were indeed inadequately described. Various methods are reported and used in the literature to select a cut-off point which defines two groups with different risks for a continuously measured marker (e.g. the 6MWT). Only two of them are based on the maximization of the true classification rate, i.e. the Youden’s Index and the concordance probability (*Unal I. (2017). Defining an Optimal Cut-Point Value in ROC Analysis: An Alternative Approach. Computational and mathematical methods in medicine, 2017, 3762651.* & *Kim SJ, Myong JP, Suh H, Lee KE, Youn YK. Optimal Cutoff Age for Predicting Mortality Associated with Differentiated Thyroid Cancer. PLoS One. 2015;10(6):e0130848. Published 2015 Jun 23.*). The reviewer is right that other methods, e.g. the point closest to the ROC curve, are inferior and inappropriate to the above mentioned methods and induce a considerable risk of bias (*Perkins NJ, Schisterman EF. The inconsistency of "optimal" cutpoints obtained using two criteria based on the receiver operating characteristic curve. Am J Epidemiol. 2006;163(7):670-675.*). In our manuscript, we only used the ROC-curve to identify potentially relevant outcome measures (AUC above 0.7) (*Mandrekar JN. Receiver operating characteristic curve in diagnostic test assessment. J Thorac Oncol. 2010 Sep;5(9):1315-6.*). Subsequently, we used the Youden’s index to identify the most optimal cut-off point for our population. In the previous versions of the manuscript, we wrongly reported that a ROC-analysis (suggesting the ‘closest point to 0,1’-method) was used to determine a cut-off point for 6MWT; this statement was incorrected. We corrected this information.

The following sentences were adapted in the revised version of the manuscript:

Results, pages 8-9, lines 166-171: “We performed a receiver operating characteristic (ROC) analysis to identify relevant outcome measures (i.e. measures with an area under the curve (AUC) above 0.7) Subsequently, based on the Youden’s index, a 298 m cut-off point (specificity = 0.583; sensitivity = 0.889) on the 6MWT test was established and used in the survival analysis (Fig 1).”

We also agree with the reviewer that some bias could be present because we used data to calculate both a cut-off point and hazard ratio’s. Additionally, residual bias may be present due to censoring. Therefore, we added these concerns to the limitations of the manuscript. Additionally, we also included a statement in future research perspectives on the recommendation to validate and examine this cut-off point in other cohorts of patients on haemodialysis.

The following sentences were adapted in the revised version of the manuscript:

Discussion, page 18, lines 378-382: “Fourth, mortality data were used to calculate both a cut-off point and hazard ratio’s, potentially bringing some bias to the analysis. In addition, bias due to censoring might still be present despite the use of a competing risks analysis. Therefore, our data should be interpreted with caution.”

Conclusions and guidelines for further research, page 19, lines 394-396: “Additionally future research should try to validate and examine the cut-off point in other cohorts of haemodialysis patients as well as to examine the true clinical importance of the associations reported in the present study.”

**4) The number of units reported in a continuous factor, as it affects a survival outcome, should correspond to a meaningful effect on mortality. As such the effect on mortality with respect to 10 or more meters increase in 6MWT should be reported. The resulting hazard ratio (about 0.96) would have more meaning clinically.**

We agree with the reviewer that the establishment of a Minimal Clinically Important Difference (MCID) would be interesting. Such a MCID would be of added value to clinicians in their interpretation of the 6MWT as well as the change of 6MWT over time. However, such a MCID is preferably examined via a longitudinal study with multiple assessments over time (*Crosby RD, Kolotkin RL, Williams GR. Defining clinically meaningful change in health-related quality of life. J Clin Epidemiol. 2003 May;56(5):395-407.*). Therefore, our data is not appropriate to be used in a MCID-analysis. Nevertheless, our data indicate that measures of physical function are associated with mortality and future research should examine the true (clinical) importance of this association. To meet with the reviewers suggestion, we added a statement to Future Research Perspectives.

The following sentence was added to the revised version of the manuscript:

Conclusions and guidelines for further research, page 19, lines 394-396: “Additionally future research should try to validate and examine the cut-off point in other cohorts of haemodialysis patients as well as to examine the true clinical importance of the associations reported in the present study.”

**5) Line 88: "Predicting" should be "predictive".**

We corrected the sentence.

The following sentence was adapted in the revised version of Materials and methods:

Materials and methods, page 5, lines 87-89: “This longitudinal study was part of a project to identify potential (predictive) biomarkers based on functional capacity, nutritional status and/or QoL in patients on dialysis (registration number on clinicaltrial.gov: NCT03910426).”

**6) When reporting a variable, if the standard deviation exceeds the mean, there is a strong indication of a skew in the distribution. As a result, the 25% and 75% quantiles should be reported. This applies specifically to the variables "Dialysis vintage" and "Davies comorbidity score" in Table 1.**

Thank you for this remark. We performed the requested adaptations.

The following information was adapted in the revised version of the manuscript:

Results, pages 10-11, lines 210-213: “

Table 1. Patient characteristics.

| Variable | n = 117 |
| --- | --- |
| Dialysis vintage (months) | 36 [11; 55] |
| Davies comorbidity score (0-7) | 2 [1; 3] |

Data are presented as mean ± standard deviation, as number (percentage) and as mean [25th; 75th] quantiles. BMI, body mass index; CVD, cardiovascular disease; VAS, visual analogue scale; 6MWT, 6-minute walking test”

**7) More description is needed for the randomization scheme and the variable selection procedure. For the randomization scheme, how was a particular randomization assigned to each patient? Was balance accounted for or assessed? Was the effect of ordering considered in the statistical modeling? With respect to variable selection, what criterion (e.g., AIC or BIC) was used to choose the appropriate model? It appears that the variable selection procedure did not actually take out any variables for the final multivariable models; is that the case?**

We randomized the sequence of physical examinations by using six opaque envelopes. Each envelope containing an assessment of physical function, i.e. (1) quadriceps strength, (2) handgrip strength, (3) sit-to-stand test, (4) six-minute walking test, (5) Tinetti test, and (6) the FICSIT. The patients were asked to order the opaque envelopes randomly and the tests were performed as such.

This information was added to the revised version of the manuscript:

Materials and methods, page 6, lines 96-99: “The sequence of physical examinations was randomized using six opaque envelopes (one for each assessment of physical function, i.e. quadriceps strength, handgrip strength, sit-to-stand test, six-minute walking test, Tinetti test, and the FICSIT) and the patients were asked to randomly order the envelopes.”

Balance was assessed via the FICSIT test. The ‘Frailty and Injuries: Cooperative Studies of Intervention Techniques-4’ is a static balance test commonly used for a variety of elderly populations (*Rossiter-Fornoff J, Wolf S, Wolfson L, Buchner D. A cross-sectional validation study of the FICSIT common data base static balance measures. Frailty and Injuries: Cooperative Studies of Intervention Techniques. Journals of Gerontology Series A1995. p. 291-7* & *Blankevoort CG, van Heuvelen MJ, Scherder EJ. Reliability of six physical performance tests in older people with dementia. Phys Ther. 2013;93(1):69-78*). To my knowledge, the scale has not been studied in patients on haemodialysis and no clear cut-off points were reported in the literature. Patients were asked to take a sequence of 7 positions with progressive difficulty but without the use of any assistive devices, the positions were: (1-2) parallel stance with feet closely together (eyes open and closed), (3-4) semi-tandem stance (eyes open and closed), (5-6) tandem stance (eyes open and closed) and (7) unipedal stance with the preferred leg. Patients were scored on each position to a 5-point scale with a total maximum score of 28, based on test-time (up to 10 seconds) and need for feedback or aid. If patients were not able to complete a subtest, the assessment of the FICSIT-4 stopped (*Rossiter-Fornoff J, Wolf S, Wolfson L, Buchner D. A cross-sectional validation study of the FICSIT common data base static balance measures. Frailty and Injuries: Cooperative Studies of Intervention Techniques. Journals of Gerontology Series A1995. p. 291-7*).

The effect of ordering was not examined in the statistical analysis. Only measures that showed significant associations in the univariate model were introduced in the multivariate model. No stepwise method was used in the present study. This was wrongfully reported in Materials and methods. We apologize for this mistake, which happened because a stepwise backward regression model was used previously in other published articles. Although a stepwise regression model is superior to the method that we used in the present study, we were not able to combine this statistical method with the competing risks regression model. We added this limitation to the discussion of the manuscript.

The following sentence was added to the revised Discussion:

Discussion, page 18, lines 381-382: “Fifth, the variable selection in the multivariate analysis was based on the univariate analysis and not on a stepwise selection model.”

**8) Table 3 should be described more specifically. What subset of the population was used? What was the criterion of selection? How large was that subset?**

Thank you for this suggestion to make the manuscript more transparent. The same criteria were used in Table 3 as in Table 1 to identify impairments in measures of physical function of patients. More information of the subset of the population was added to Table 3 (i.e. the ratio of patients with and without impaired physical function).

The following information was added to Table 3:

Results, page 12, lines 236-237: “

Table 3. Unadjusted competing risks regression model for mortality by the presence of impairments in physical performance.

|  | Mortality (event = death) | | | |
| --- | --- | --- | --- | --- |
| Impairments (impaired (n); not impaired (n)) | Univariate model | | Multivariate model | |
|  | HR | p-value | HR | p-value |
| Quadriceps strength (108; 9) | 5.291 | 0.045 | 2.611 | 0.293 |
| Handgrip strength (43; 74) | 2.433 | 0.003 | 1.721 | 0.079 |
| Sit-to-Stand (81; 36) | 4.566 | 0.001 | 1.403 | 0.573 |
| Tinetti (58; 59) | 3.215 | <0.001 | 0.001 | 0.778 |
| DFRI (87; 30) | 20.408 | 0.003 | / | NA |
| 6MWT (80; 37) | 7.407 | <0.001 | 5.882 | 0.002 |

”

**9) Since modeling using both the cutoff version of 6MWT and the continuous version of 6MWT were used in the modeling, the write-up in the Results of the use of these variables should be more careful. In particular, the description on lines 214-221 jumps between the two characterizations in a way that could be confusing to the reader.**

We improved the transparency of the manuscript by adding more details on the type of the data in this section.

The following sentences were adapted in the revised version of the manuscript:

Results, page 11, lines 221-229: “A walking distance of less than 298 meters (specificity = 0.583; sensitivity = 0.889, Fig 1) was associated with an increased mortality risk (HR = 7.4, p=0.045). Only exercise capacity as a continuous variable was retained as a predictor of survival in the multivariate analyses (6MWT (m): HR = 0.997, p=0.005; impaired 6MWT: HR = 5.880, p=0.002). After adjusting for age, sex, comorbidity and dialysis vintage, each increase on the 6MWT (m) was associated with a 0.4% decrease in mortality risk (HR = 0.996, 95%CI [0.994; 0.998], p<0.001, Table 4). The results of the adjusted survival analysis for the 298 meters walking distance cut-off point (i.e. the categorical analysis of exercise capacity) were similar to the results of the unadjusted analysis (HR = 7.576, p<0.001, Table 5).”

# REVIEWER #2

**Thank you for your effort to revise your manuscript. You have done great effort and responded to suggested comments. I have no further comments on your work.**

Thank you for the constructive remarks.
