# Supplementary material for: The importance of physical performance in the assessment of patients on haemodialysis: A survival analysis
Source: PLoS One. 2022 May 19;17(5):e0268115. doi: 10.1371/journal.pone.0268115 (PMC9119466; doi:10.1371/journal.pone.0268115)
Supplement: Attachment — Submitted filename: Response to Reviewers.docx [file pone.0268115.s007.docx]

**Revision: manuscript PONE-D-20-37730R2**

**The importance of physical performance in the assessment of patients on haemodialysis: a survival analysis**

Dear Editor,

We would like to thank you again for the opportunity to improve our work and to give us the chance to resubmit the manuscript to PLOS ONE. We are grateful for the editorial and reviewer comments and thank them for their appreciation.

The formulated comments are well taken; below you will find point-by-point responses to the comments and questions raised in the decision letter. Stated citations (page; line) refer to the revised version of the manuscript with track changes.

Yours sincerely,

Karsten Vanden Wyngaert

1. EDITORIAL COMMENTS

# JOURNAL REQUIREMENTS AND EDITOR COMMENTS

**1) Please review your reference list to ensure that it is complete and correct. If you have cited papers that have been retracted, please include the rationale for doing so in the manuscript text, or remove these references and replace them with relevant current references. Any changes to the reference list should be mentioned in the rebuttal letter that accompanies your revised manuscript. If you need to cite a retracted article, indicate the article’s retracted status in the References list and also include a citation and full reference for the retraction notice.**

We checked the references and updated those that could be updated. The following 14 citations in the old version of the manuscript were updated: 2, 9, 27, 29, 30, 32, 33, 34, 35, 45, 47, 48, 50, and 51.

The following citations were updated and changed in revised version of the manuscript:

References, page 21: “2. Van Craenenbroeck AH, Van Craenenbroeck EM, Van Ackeren K, Hoymans VY, Verpooten GA, Vrints CJ, et al. Impaired vascular function contributes to exercise intolerance in chronic kidney disease. Nephrology, dialysis, transplantation : official publication of the European Dialysis and Transplant Association - European Renal Association. 2016;31(12):2064-72. Epub 2016/08/20. doi: 10.1093/ndt/gfw303. PubMed PMID: 27540045.”

References, page 21: “9. Ju A, Unruh M, Davison S, Dapueto J, Dew MA, Fluck R, et al. Establishing a Core Outcome Measure for Fatigue in Patients on Hemodialysis: A Standardized Outcomes in Nephrology-Hemodialysis (SONG-HD) Consensus Workshop Report. American journal of kidney diseases : the official journal of the National Kidney Foundation. 2018;72(1):104-12. Epub 2018/03/20. doi: 10.1053/j.ajkd.2017.12.018. PubMed PMID: 29551585.”

References, page 22: “27. Rossiter-Fornoff JE, Wolf SL, Wolfson LI, Buchner DM. A cross-sectional validation study of the FICSIT common data base static balance measures. Frailty and Injuries: Cooperative Studies of Intervention Techniques. The journals of gerontology Series A, Biological sciences and medical sciences. 1995;50(6):M291-7. Epub 1995/11/01. doi: 10.1093/gerona/50a.6.m291. PubMed PMID: 7583799.”

References, page 22: “29. Prinja S, Gupta N, Verma R. Censoring in clinical trials: review of survival analysis techniques. Indian J Community Med. 2010;35(2):217-21. Epub 2010/10/06. doi: 10.4103/0970-0218.66859. PubMed PMID: 20922095; PubMed Central PMCID: PMCPMC2940174.”

References, page 22: “30. Davies SJ, Phillips L, Naish PF, Russell GI. Quantifying comorbidity in peritoneal dialysis patients and its relationship to other predictors of survival. Nephrology, dialysis, transplantation : official publication of the European Dialysis and Transplant Association - European Renal Association. 2002;17(6):1085-92. Epub 2002/05/29. doi: 10.1093/ndt/17.6.1085. PubMed PMID: 12032201.”

References, page 22: “32. Kramer A, Boenink R, Stel VS, Santiuste de Pablos C, Tomović F, Golan E, et al. The ERA-EDTA Registry Annual Report 2018: a summary. Clinical kidney journal. 2021;14(1):107-23.”

References, page 22: “33. Masnoon N, Shakib S, Kalisch-Ellett L, Caughey GE. What is polypharmacy? A systematic review of definitions. BMC Geriatr. 2017;17(1):230. Epub 2017/10/12. doi: 10.1186/s12877-017-0621-2. PubMed PMID: 29017448; PubMed Central PMCID: PMCPMC5635569.”

References, page 22: “34. Van Craenenbroeck AH, Van Craenenbroeck EM, Kouidi E, Vrints CJ, Couttenye MM, Conraads VM. Vascular effects of exercise training in CKD: current evidence and pathophysiological mechanisms. Clinical journal of the American Society of Nephrology : CJASN. 2014;9(7):1305-18. Epub 2014/05/15. doi: 10.2215/CJN.13031213. PubMed PMID: 24832091.”

References, page 22: “35. Collins AJ, Foley RN, Herzog C, Chavers BM, Gilbertson D, Ishani A, et al. Excerpts From the US Renal Data System 2009 Annual Data Report. American Journal of Kidney Diseases. 2010;55(1):A6-A7. doi: 10.1053/j.ajkd.2009.10.009.”

References, page 22: “45. Johansen KL, Chertow GM, Jin C, Kutner NG. Significance of Frailty among Dialysis Patients. Journal of the American Society of Nephrology. 2007;18(11):2960-7. doi: 10.1681/asn.2007020221.”

References, page 22: “47. Pritchard JM, Kennedy CC, Karampatos S, Ioannidis G, Misiaszek B, Marr S, et al. Measuring frailty in clinical practice: a comparison of physical frailty assessment methods in a geriatric out-patient clinic. BMC Geriatr. 2017;17(1):264. doi: 10.1186/s12877-017-0623-0.”

References, page 22: “49. Johansen KL, Delgado C, Kaysen GA, Chertow GM, Chiang J, Dalrymple LS, et al. Frailty Among Patients Receiving Hemodialysis: Evolution of Components and Associations With Mortality. The journals of gerontology Series A, Biological sciences and medical sciences. 2019;74(3):380-6. Epub 2018/09/08. doi: 10.1093/gerona/gly206. PubMed PMID: 30192916; PubMed Central PMCID: PMCPMC6376100.”

References, page 22: “50. Qazi HA, Chen H, Zhu M. Factors influencing dialysis withdrawal: a scoping review. BMC nephrology. 2018;19(1):96. Epub 2018/04/28. doi: 10.1186/s12882-018-0894-5. PubMed PMID: 29699499; PubMed Central PMCID: PMCPMC5921369.”

References, page 22: “51. van Loon IN, Goto NA, Boereboom FTJ, Verhaar MC, Bots ML, Hamaker ME. Quality of life after the initiation of dialysis or maximal conservative management in elderly patients: a longitudinal analysis of the Geriatric assessment in OLder patients starting Dialysis (GOLD) study. BMC Nephrology. 2019;20(1):108. doi: 10.1186/s12882-019-1268-3.”

2. RESPONSE TO REVIEWERS

# REVIEWER #1

**The authors are to be congratulated on addressing a great many issues related to the technical and data analytic aspects of this article. However, several important issues have not been fully addressed.**

Thank you for this comment. We are happy that most of the items were sufficiently addressed.

**1) Lines 167-169 Receiver operating character analysis and specificity/sensitivity analyses are diagnostic functions of a generalized regression model. From the text, it can be inferred which predictors -- 6MWT, for instance -- are being used, but the authors have still not specified which outcome is associated with this modeling. Is it death? What categorical outcome is being used to generate these results?**
Thank you for clarifying this previous comment. The categorical outcome measure was indeed mortality (death). Because this information was lacking, we added this information to the sentence.

The following sentence was adapted in the revised version of the manuscript:

Materials and methods, page 9, lines 167-169: “We performed a receiver operating characteristic (ROC) analysis to identify relevant outcome measures (i.e. measures with an area under the curve (AUC) above 0.7) with regard to mortality.”

**2) Several issues remain with the Statistical Analysis section. 163 For which reported outcome (in the text) was the chi-square test used?**

Thank you for addressing this error. If we would have examined the differences in incidence of death between groups, we would have used the chi-square test. However, the chi-square to examine such a difference was indeed not used. We deleted this sentence.

**4) Lines 255-284 For the comorbidity analysis, the outcomes seem to be ordinal categories based on the Davies comorbidity index. Was this the case? Please specificy.**

The reviewer is correct. The Davies comorbidity index is an ordinal variable, which can identify patients with a low, medium or high mortality risk. However, the results reported in Tables 6 and 7 are based on the Davies Comorbidity scale (0-7), which is the sum of active (relevant) comorbid conditions in a patient. We clarified the results by adding this information.

The following information was added to the revised version of results:

Results, page 13, lines 262-265: “Similar to the survival analysis, all domains of physical performance were associated with the degree of morbidity based on the Davies comorbidity scale (0-7), albeit only the 6MWT remained relevant in the multivariate analysis (R^2^ = 0.145, p=0.012, Table 6).”

Results, page 13, lines 265-266: “Adjusted for age, sex and dialysis vintage, the 6MWT explained 20.8% of the variance in the Davies comorbidity scale (p<0.001, Table 7).”

Results, page 14, lines 268-269: “Table 6. Unadjusted general linear model of the different measures of physical performance on comorbidity (Davies comorbidity scale 0-7).”

Results, page 14, lines 273-274: “Table 7. Adjusted general linear model of the 6MWT on comorbidity (Davies comorbidity scale 0-7).”

**5) Additionally, please provide a more detailed description of the modeling that was done for this analysis. A generalized linear model (or a "general linear model") is not a sufficient description.**

Thank you for this remark. Because we used the comorbidity scale and not the index, we considered the scale as a continuous variable and, therefore, used a linear model. The reviewer is correct that a different model should have been used when the index (low, medium, high risk classification) was used. Nevertheless, we are of the opinion that a general linear model was appropriate to examine the association between comorbidity (as a continuous variable) and measures of physical performance.
