# Supplementary material for: The importance of physical performance in the assessment of patients on haemodialysis: A survival analysis
Source: PLoS One. 2022 May 19;17(5):e0268115. doi: 10.1371/journal.pone.0268115 (PMC9119466; doi:10.1371/journal.pone.0268115)
Supplement: Attachment — Submitted filename: Response to Reviewers.docx [file pone.0268115.s008.docx]

**Revision: manuscript PONE-D-20-37730R3**

**The importance of physical performance in the assessment of patients on haemodialysis: a survival analysis**

Dear Editor,

We would like to thank you again for the opportunity to improve our work and to give us the chance to resubmit the manuscript to PLOS ONE.

The formulated comment is well taken; we changed the term ‘general linear model’ to ‘least squares linear regression model’ as requested by the reviewer.

We would like to thank you for the extensive and patient guidance through the reviewing process.

Yours sincerely,

Karsten Vanden Wyngaert

1. RESPONSE TO REVIEWERS

# REVIEWER #1

**The authors are to be congratulated on their responses to this and previous revisions. The responses and subsequent revisions have done much to improve the clarity of the analysis description.**

Thank you for the constructive suggestions and feedback.

**There continues to be one minor comment: Instead of the term "general linear model" (which can easily be confused with a "generalized linear model"), the term "least squares linear regression model" is preferred. Unless there is some reason not described in previous revisions, the term "general linear model" should be avoided in favor of preferred standard terminology.**

Thank you for this suggestion, which will improve the transparency of our manuscript. We adapted the term “general linear model” as requested.

The following sentences were adapted in the revised version of the manuscript:

Abstract, page 2, lines 26-28: “Data were analysed by least squares linear regression models and competing risks survival hazard models.”

Materials and methods, page 9, lines 182-184: “Next, least squares linear regression models were used to evaluate the association between hospitalisation and comorbidity on the one hand and the different measures of physical performance on the other.”

Results, page 13, lines 267-269: “Table 6. Unadjusted least squares linear regression model of the different measures of physical performance on comorbidity (Davies comorbidity scale 0-7).”

Results, page 14, lines 273-274: “Table 7. Adjusted least squares linear regression model of the 6MWT on comorbidity (Davies comorbidity scale 0-7).”

Results, page 14, lines 283-284: “Table 8. Least squares linear regression model of the different measures of physical performance on 1-year hospitalisation (days/year).
